# Epidemiology and Management of Foreign Bodies in the Hand: Pakistani Perspective

**Published:** 2014-01

**Authors:** Muhammad Saaiq

**Affiliations:** Department of Plastic Surgery, Burn Care Center, Pakistan Institute of Medical Sciences (PIMS), Islamabad, Pakistan

**Keywords:** Foreign body, Hand, Penetrating, Diagnosis

## Abstract

**BACKGROUND:**

Penetrating and impalement injuries of the hand and fingers are one of the commonest presentations at the hospital’s emergency rooms. This study assessed the characteristics of patients who suffered foreign body injuries to the hands and documented the pattern of diagnosis and management at a specialist plastic surgical facility.

**METHODS:**

The study was conducted at the Department of Plastic and Reconstructive Surgery, Pakistan Institute of Medical Sciences (PIMS), Islamabad over a period of six years

(i.e. from September 1, 2007 to July 31, 2013). All adult patients (37 subjects) of either gender who were managed for hand foreign bodies during the study period were included by convenience sampling technique. The demographic profile of the patients, cause of injury, type of foreign body, occupation of the patient, diagnostic yield of plain x-rays, type of procedure undertaken for retrieval of foreign body, and complications were all recorded on a form. A follow-up of three months was done.

**RESULTS:**

Eighteen (48.64%) were males while 51.35% (n=19) were female. The mean age was 26.78±9.94 years. The commonest sufferers were housewives 29.72% (n=11). Majority of patients (n=16; 43.24%) presented on day 3 (i.e. >48-72 hours), among the injury causing mechanisms, the commonest were accidents with sewing machines 45.94% (n=17) and sewing machine needles 45.94% (n=17) were the commonest foreign bodies observed. The plain x-ray hands reveled the diagnosis in all patients except those with wooden foreign bodies (n=3; 8.10%). All patients had successful surgical exploration and retrieval of the foreign bodies under local anesthesia and tourniquet control. In two cases, image intensifier was employed to locate the foreign bodies per-operatively. Wound infection was found in 0.8% (n=4) patients, all of whom were managed successfully with oral antibiotics. None of patients had hospitalization. All patients were fine at 3 months follow up.

**CONCLUSIONS:**

Surgical exploration and careful retrieval under local anesthesia and tourniquet control suffice as the definitive treatment. Rarely intra-operative image intensifier is needed to locate foreign bodies per-operatively.

## INTRODUCTION

Hands constitute one of the most frequently used organs of the human body. Owing to their use as the frontline performers in executing daily activities, they are vulnerable to sustain various traumatic insults while at work.^1^ Penetrating and impalement injuries of the hand and fingers are one of the commonest presentations at the hospital’s emergency rooms both in the developed as well as developing countries. Foreign body injuries should be suspected in all such cases.^2^^,^^3^

If a foreign body is missed initially, it may remain asymptomatic for prolonged periods of time or may give rise to a variety of inflammatory, allergic and infectious complications such as formation of foreign body granulomas, pyogenic granulomas, abscess, chronic discharging wound, and problems with underlying structures like bones, tendons, blood vessels and nerves. In order to avert these complications, the hand foreign bodies are best removed surgically.^4^^-^^7^

The present study was undertaken to assess the clinical presentation, diagnosis and management of hand foreign bodies in a plastic surgical setting, and hence evolve an actionable evidence base that would better guide management of future patients sustaining such injuries.

## MATERIALS AND METHODS

This descriptive case series study was carried out at the Department of Plastic Surgery, Pakistan Institute of Medical Sciences (PIMS), Islamabad over a period of six years (i.e. from September 1, 2007 to July 31, 2013). All adult patients of either gender who were managed for isolated hand foreign body injuries during the study period were included by convenience sampling technique. Patients with other associated hand injuries such as fractures, soft tissue loss, tendon injury and those with associated injuries to other parts of the body were excluded. 

The initial assessment was made by history, thorough hand examination and plain X-rays hand (anteroposterior and lateral views) in all patients. Tetanus prophylaxis was routinely employed if there was no booster dose of tetanus immunization within 10 years. Passive immunization with tetanus immune globulin was also ensured in cases of contaminated wounds with no known or complete history of tetanus immunization.

All patients were managed on outdoor basis. Surgical exploration and careful retrieval under local anesthesia and tourniquet control was undertaken under aseptic precautions. Cases where surgical exploration did not identify the foreign body, image intensifier was employed to locate the foreign bodies per-operatively. The demographic profile of the patients, cause of injury, occupation of the patient, type of foreign body, diagnostic yield of plain x-rays, type of procedure undertaken for retrieval of foreign body, any complications were all recorded on a form. A follow-up of three months was done. 

The data were analyzed by SPSS software (Version 17, Chicago, IL, USA) and various descriptive statistics were used to calculate frequencies, percentages, means and standard deviation. The numerical data such as age were expressed as mean±standard deviation while the categorical data such as the causes of hand foreign bodies and types of foreign bodies were expressed as frequency and percentages.

## RESULTS

Out of a total of 37 patients, 48.64% (n=18) were males while 51.35% (n=19) were female. The mean age was 26.78±9.94 years. Majority of patients (78.36%) were less than 30 years of age. Right hand was dominant among all patients. The commonest sufferer was housewives 29.72% (n=11). Occupations of the remainder of the patients included tailors 16.21% (n=6); hardware dealers/welders, 13.50% (n=5); nursing, 5.40% (n=2); farmers, 2.70% (n=1); and woodworkers, manual workers, students and office workers, 8.10% (n=3) each.

Majority of the patients (n=16; 43.24%) presented on day 3 (i.e. >48-72 hours), while 7 (18.91%) patients presented within 24 hours, 5 (13.51%) patients within 48 hours and 9 (24.32%) patients after 72 hours. Among the injury causing mechanisms, the commonest were accidents with sewing machines, 45.94% (n=17). The remainder included hardware dealing/welding, 13.50% (n=5); road traffic accidents and manual labor works, 10.80% (n=4) each; woodworks, 8.10% (n=3); breaking medicine vials for injection, 5.40% (n=2); fire arm injury and animal spike, 2.70% (n=1) each.

Among the foreign bodies observed, the commonest were sewing machine needles, 45.94% (n=17); followed by metallic fragments, 18.91% (n=7); glass pieces, 16.21% (n=6); wood splinters and stones/gravels, 8.10% (n=3) each; and animal spine, 2.70% (n=1). The plain x-ray hands reveled the diagnosis in all patients except those with wooden foreign bodies (n=3; 8.10%).

All patients had successful surgical exploration and retrieval of the foreign bodies. In two cases, image intensifier was employed to locate the foreign bodies per-operatively. Wound infection was found in 10.8% (n=4) patients, all of whom were managed successfully with oral antibiotics. None of our patients had hospitalization. All patients were fine at 03 months follow up.

## DISCUSSION

In our study, we had almost equal numbers of patients from either gender. Our finding contrasts to the observation of Salati *et al*.^8^ and Mohammadi *et al*.^9^ who reported a gender difference in foreign body injury patterns with predominant involvement of males. The difference is probable due to more frequent involvement of our women in sewing activities which has earned the status of a cottage industry in our villages and towns. 

Majority of our patients were relatively young. Other published studies have also reported more frequent involvement of relatively younger patients sustaining such injuries.^5^^-^^9^ By and large, the risk of various hand injuries tend to decline with increasing age and growing experience of the workers. 

Majority of our patients presented after 48 hours of sustaining the injury. Our observation contrasts to that of Levine *et al.*^10^ who reported majority of their patients presenting within 48 hours. In our series, finger penetration with sewing machine needle was the most common form of foreign body injury to the hand. Published studies have reported a variety of other causes of foreign bodies in the hand.^5^^-^^7^^,^^10^ The published literature has also reported some rare causes of foreign body injuries of the hand. For instance, patients undergoing instrumentation, surgery or other therapeutic interventions may sustain an iatrogenic injury involving foreign bodies. Some individuals practicing wizardry may insert wires, paper clips etc in themselves.^11^^,^^12^

In our series, we did not find any case of self injury that resulted in foreign body injury to the hand. Contrary to this, there is growing recognition of self-injury characterized by the deliberate and direct destruction or alteration of body tissues without suicidal intent. It is estimated that in the United States, 4% of the general population and 13-23% of the adolescents report a history of non-suicidal self-injury.^13^^-^^15^ Such self-inflicted injuries if any in our part of the world are not yet reported in the local published literature.

In our study, one of our patients who were a farmer by profession presented with hand foreign body secondary to being attacked by a spiny anteater while working in his farms. We could not find any such case previously reported in the published literature. In our study we performed plain x-rays in all our patients. All metallic foreign bodies confirmed on surgical exploration were detected on pre-operative plain x-rays. Our observation conforms to that of several other reported studies. For instance, Hunter TB *et al.*^16^ have observed that all metallic foreign bodies except aluminum, are radio-opaque on plain radiographs. 

In our series, glass foreign bodies were the second largest type of injury source. Plain x-rays detected all these injuries. Our observation conforms to other reported studies. Fornage *et al.*^17^ have reported that any piece of glass 1–2 mm or larger should generally be visible on x-rays. In our series, there were three cases of wooden foreign bodies, however none of them could be detected on pre-operative x-rays and all of them were confirmed on surgical exploration only. Peterson *et al.*^18^ who reported a series of 12 cases of retained foreign bodies also observed failure of plain x-rays to reveal the diagnosis. Other published studies have also reported x-rays to be poor in diagnosing wooden foreign bodies. Radiographs may reveal a wooden foreign body in only up to 15% of patients. The wooden foreign bodies are usually radiolucent, associated with gas in the matrix. However, the small size of the foreign body often is not sufficient to create an appreciable radiolucency. Wood usually shows a linear hypo-intense signal on MR imaging with an associated inflammatory mass. CT typically shows the retained wood as a linear area of increased attenuation, which is best seen on wide window settings. Sonography has proved the most useful modality, easily identifying the retained wood as a linear echogenic focus with marked acoustic shadowing.^18^^-^^20^

We did not employ ultrasound as a diagnostic modality in any of our patients; however, the published literature reveals a growing trend towards more frequent use of high frequency ultrasound for both diagnosis and management of hand foreign bodies.^21^^-^^23^

Our study had some limitations. It was a single centered study. Randomization and blinding of the patients or treating doctors were not possible and so observer bias could not be eliminated completely. We could not evaluate cosmetic or long term functional results among the patients. 

Our study should prompt other similar local studies and hence allow more meaningful comparison of results in our own population. We recommend the conduct of a multicentre local study to confirm and improve upon our results. Also a local study may be conducted to evaluate the overall cost of management and loss of working days as a consequence of such injuries. 

Given the evidence base, we recommend that educational program should be launched to create awareness among public about the significant consequences of these injuries. Occupational safety protocols for the at-risk-individuals at different workplaces should be devised and implemented. The primary focus of such protocols should be the machine related work environments, woodworks and glass industry. 

Hand foreign bodies are not uncommon in cases of penetrating and impalement injuries of the hand. Majority of the sufferers are young individuals of either gender and present with history of injury mechanisms that suggest the presumptive diagnosis of foreign bodies. Sewing machine needles are the commonest foreign bodies encountered in our population. Plain x-rays reliably diagnose and locate metallic, glass and stony foreign bodies, however wooden foreign bodies are often not revealed by plain x-rays. Surgical exploration and careful retrieval under local anesthesia and tourniquet control suffice as the definitive treatment. Rarely intra-operative image intensifier is needed to locate foreign bodies per-operatively.

## Footenote

Please cite this paper as:

Saaiq M. Epidemiology and Management of Foreign Bodies in the Hand: Pakistani Perspective. World J Plast Surg 2014;3(1):13-17.
